# Machine learning prediction of feeding intolerance in preterm infants: a pre-feeding risk stratification model

**DOI:** 10.3389/fped.2025.1646973

**Published:** 2025-09-05

**Authors:** Gai Mao, Yue Li, Min Li, Jin Wang, Ying Li

**Affiliations:** ^1^ Department of Traditional Chinese Medicine, Capital Center for Children's Health, Capital Medical University, Capital Institute of Pediatrics, Beijing, China; ^2^ Department of Neonatology, Capital Center for Children's Health, Capital Medical University, Capital Institute of Pediatrics, Beijing, China

**Keywords:** feeding intolerance, preterm infants, machine learning, risk prediction, adaboost, early intervention

## Abstract

**Background:**

Feeding intolerance (FI) represents a prevalent and serious complication in preterm infants, contributing to delayed enteral nutrition, prolonged hospitalization, and increased morbidity. Early identification of high-risk infants remains challenging due to limited predictive tools available before feeding initiation.

**Methods:**

We conducted a retrospective cohort study of 402 preterm infants (<37 weeks gestational age) admitted between January 2023 and May 2024. Clinical data collected at admission underwent feature selection using cross-validated LASSO regression. Eleven machine learning algorithms were systematically compared using accuracy, area under the receiver operating characteristic curve (AUC), sensitivity, and specificity. Clinical utility was assessed through decision curve analysis (DCA).

**Results:**

FI developed in 199 (49.5%) infants. Significant between-group differences were observed for birth weight, gestational age, time to first feeding, fetal distress, multiple gestation, prenatal dexamethasone exposure, neonatal infection, respiratory distress, and invasive mechanical ventilation (all *P* < 0.01). LASSO regression identified 14 optimal predictive variables. Among tested algorithms, AdaBoost demonstrated superior performance [accuracy: 0.957; AUC: 0.964 (95% CI: 0.929–1.000); sensitivity: 0.957; specificity: 0.958]. DCA confirmed greater net clinical benefit compared to “treat all” or “treat none” strategies. An interactive clinical decision support tool was developed for practical implementation.

**Conclusions:**

The proposed machine learning model accurately predicts feeding intolerance before first feeding using 14 routinely collected clinical variables. This approach enables early risk stratification and may improve clinical outcomes through timely intervention. External validation in multicenter cohorts is warranted to confirm generalizability.

## Introduction

Feeding intolerance (FI) is a common and clinically significant complication among preterm infants, with global incidence estimates ranging from 16%–29% (1). FI, defined as the inability to tolerate enteral nutrition, is characterized by increased gastric residuals, vomiting, abdominal distension, and the need for feeding interruption or delay. The clinical consequences of FI are substantial, including higher rates of necrotizing enterocolitis (NEC), prolonged dependence on parenteral nutrition, impaired growth, and adverse neurodevelopmental outcomes (2). The pathophysiology of FI is multifactorial, involving gastrointestinal dysmotility, immature digestion, impaired mucosal defense, and altered gut microbiota. Perinatal complications such as respiratory distress, infection, and intrauterine inflammation further compound this risk (3).

Despite advances in neonatal intensive care, the early identification of infants at risk for FI remains an unmet clinical need (4). International guidelines from European Society for Paediatric Gastroenterology, Hepatology and Nutrition (ESPGHAN) and the World Health Organization (WHO) emphasize the importance of early enteral feeding, the use of mother's own milk, and the minimization of feeding interruptions (5, 6). However, most guidelines do not provide robust tools for individualized risk prediction prior to feeding initiation. Conventional risk scores are often based on post-symptomatic markers and lack sufficient sensitivity or specificity for clinical utility.

Recent developments in machine learning (ML) have enabled the analysis of high-dimensional clinical data to identify complex, nonlinear relationships among multiple predictors (7). Although several ML models have demonstrated superior performance compared to traditional risk scores in predicting FI or NEC, most studies to date have utilized a limited set of variables and focused on infants already showing early symptoms (8). There is a pressing clinical need for integrative, pre-feeding risk models that leverage routinely available clinical data and can be seamlessly incorporated into clinical workflows (9).

This study aimed to develop and validate a machine learning model for the pre-feeding prediction of FI in preterm infants using routinely collected clinical variables.

## Methods

### Patient selection and study parameters

Preterm infants were enrolled from January 2023 to May 2024, with a total of 402 infants included in the study. Inclusion criteria were: (1) gestational age less than 37 weeks; (2) admission to our neonatal intensive care unit within 24 h of birth. Exclusion criteria were: (1) congenital gastrointestinal malformations, congenital genetic abnormalities, or genetic metabolic diseases; (2) development of gastrointestinal diseases such as necrotizing enterocolitis or intestinal obstruction during hospitalization; (3) participation in other clinical trials.

Feeding intolerance was diagnosed based on the following criteria: (1) gastric residual volume exceeding 50% of the previous feeding volume, accompanied by vomiting and/or abdominal distension; (2) feeding plan modifications including reduction, delay, or interruption of enteral feeding. A diagnosis of FI was established if either criterion was met.

This study was approved by the Ethics Committee of Children's Hospital Affiliated to the Capital Institute of Pediatrics (Ethics Review No. SHERLL2023071). Written informed consent was obtained from the guardians of all participating infants.

Data were collected for two categories of variables: (1) baseline characteristics including gender, gestational age, birth weight, time to first feeding, parental age, and maternal pregnancy complications; (2) neonatal factors including neonatal infection, respiratory distress after birth, and requirement for mechanical ventilation. All data were systematically organized, double-checked by two independent researchers, and entered into Excel spreadsheets.

### Data processing

Numerical variables were standardized using Z-score normalization to transform the data to a standard normal distribution with a mean of 0 and a standard deviation of 1, thereby improving model prediction performance. Pearson correlation coefficients were calculated to examine relationships between variables. For highly correlated feature pairs (correlation coefficient >0.9), we performed feature filtering by retaining only one feature from each correlated pair to mitigate multicollinearity.

A LASSO (Least Absolute Shrinkage and Selection Operator) logistic regression model was applied with cross-validation to determine the optimal regularization parameter (*λ*). Feature coefficients were evaluated, and variables with non-zero coefficients after LASSO regularization were selected for inclusion in the final model.

### Dataset splitting and model construction

The final selected feature set was randomly partitioned into training (70%) and testing (30%) sets. To enhance model robustness, random partitioning was repeated 10 times, and the split achieving the highest evaluation metrics was selected for model training and validation.

Eleven machine learning classification algorithms were employed for model construction: Logistic Regression (LR), Support Vector Machine (SVM), Random Forest (RF), Extra Trees, XGBoost, LightGBM, Naive Bayes (NB), AdaBoost, Gradient Boosting (GB), Multilayer Perceptron (MLP), and K-Nearest Neighbors (KNN). Model performance was evaluated using multiple metrics including accuracy, area under the receiver operating characteristic curve (AUC-ROC), negative predictive value (NPV), and positive predictive value (PPV). ROC curves, confusion matrices, and decision curve analysis (DCA) were generated to comprehensively assess predictive performance and clinical utility.

All analyses were conducted using Python version 3.10 (Python Software Foundation). Machine learning algorithms were implemented using open-source libraries including scikit-learn, XGBoost, and LightGBM. Data manipulation and visualization were performed using pandas, numpy, matplotlib, and seaborn libraries.

### Statistical analysis

The Kolmogorov–Smirnov test was used to assess normality of continuous variables. Normally distributed data were presented as mean ± standard deviation and compared between groups using independent *t*-tests. Non-normally distributed data were expressed as median (interquartile range) and compared using the Mann–Whitney *U* test. Categorical variables were presented as frequencies and percentages and compared using the chi-square test. A *p*-value < 0.05 was considered statistically significant.

## Results

A total of 402 preterm infants were enrolled in this study and divided into two groups: the feeding intolerance (FI) group (*n* = 199) and the feeding tolerance group (*n* = 203).

Most maternal characteristics showed no significant differences between groups, including hypertensive disorders (35 vs. 33, *P* = 0.791), gestational diabetes (37 vs. 42, *P* = 0.618), cesarean section (126 vs. 132, *P* = 0.755), maternal age (33.3 ± 4.5 vs. 33.9 ± 5.0 years, *P* = 0.348), severe preeclampsia (30 vs. 28, *P* = 0.777), maternal thyroid dysfunction (8 vs. 6, *P* = 0.598), number of pregnancies (2.0 ± 1.2 vs. 2.1 ± 1.2, *P* = 0.163), and premature rupture of membranes (71 vs. 72, *P* > 0.999). However, multiple pregnancy was significantly more common in the FI group compared to the feeding tolerance group (83 vs. 50, *P* < 0.001) (Table 1).

**Table 1:** Maternal characteristics between feeding tolerant and intolerant groups.

Parameters	Feeding intolerant group *N* = 199	Feeding tolerant group *N* = 203	*P*
Hypertensive disorders	35	33	0.791
Gestational diabetes	37	42	0.618
Cesarean section	126	132	0.755
Maternal age (years)	33.3 ± 4.5	33.9 ± 5.0	0.348
Paternal age (years)	35.0 ± 4.7	35.5 ± 6.0	0.614
Severe preeclampsia	30	28	0.777
Maternal thyroid dysfunction	8	6	0.598
Number of pregnancies	2.0 ± 1.2	2.1 ± 1.2	0.163
Premature rupture of membranes	71	72	>0.999
Multiple pregnancy	83	50	<0.001

Infants in the FI group had significantly lower mean birth weight (1,495.5 ± 502.6 g vs. 2,394.5 ± 429.0 g, *P* < 0.001) and gestational age (30.9 ± 2.8 weeks vs. 34.9 ± 1.4 weeks, *P* < 0.001) compared to the feeding tolerance group. The time to initiation of enteral feeding after birth was significantly delayed in the FI group compared to the tolerance group (66.1 ± 56.1 h vs. 15.4 ± 20.2 h, *P* < 0.001). Additionally, the FI group had significantly higher incidences of fetal distress (41 vs. 21, *P* = 0.004), intrauterine infection (113 vs. 49, *P* < 0.001), dexamethasone use (115 vs. 84, *P* < 0.001), invasive mechanical ventilation (164 vs. 41, *P* < 0.001), and respiratory distress (170 vs. 49, *P* < 0.001). No significant differences were observed for sex distribution (90/109 vs. 110/93, *P* = 0.072), placental abnormality (38 vs. 35, *P* = 0.769), or amniotic fluid abnormality (35 vs. 38, *P* = 0.630) (Table 2).

**Table 2:** Neonatal characteristics between feeding tolerant and intolerant groups.

Parameters	Feeding intolerant group *N* = 199	Feeding tolerant group *N* = 203	*P*
Sex (male/female)	90/109	110/93	0.072
Birth weight (g)	1,495.502.6	2,394.5 ± 429.0	<0.001
Gestational age (weeks)	30.9 ± 2.8	34.9 ± 1.4	<0.001
Placental abnormality	38	35	0.769
Amniotic fluid abnormality	35	38	0.630
Time to first feeding (h)	66.1 ± 56.1	15.4 ± 20.2	<0.001
Fetal distress	21	41	0.004
Intrauterine infection	49	113	<0.001
dexamethasone use	84	115	<0.001
Invasive ventilation	41	164	<0.001
Respiratory distress	49	170	<0.001

### Feature selection

**Figure 1:**
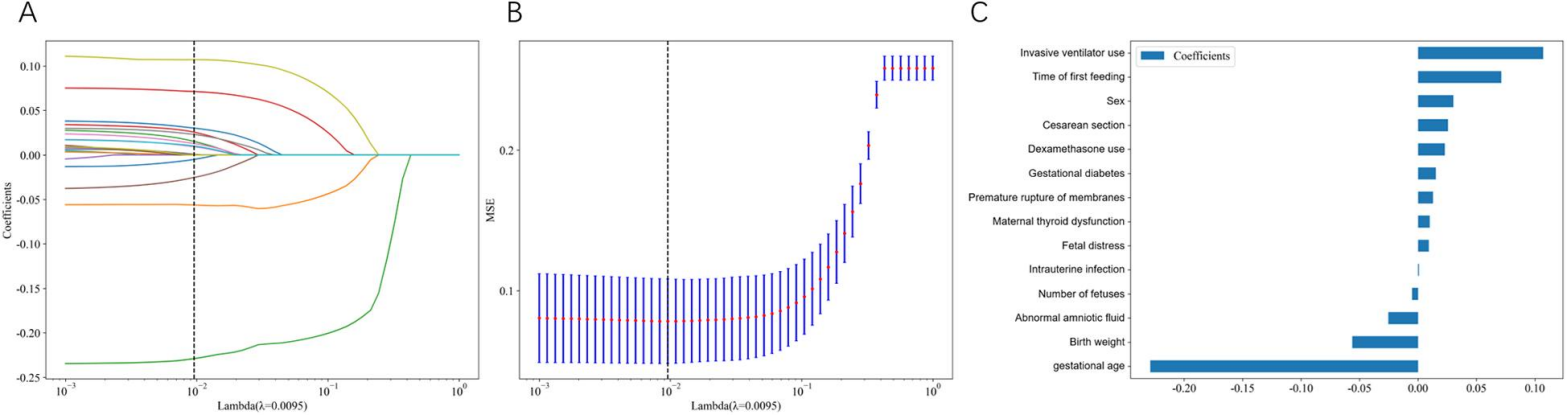
LASSO regression feature selection and model coefficients. **(A)** LASSO coefficient paths showing how feature coefficients change with varying regularization parameter *λ*. Each colored line represents a different clinical variable, and coefficients progressively shrink toward zero as *λ* increases. The vertical dashed line indicates the optimal *λ* value (0.0095) selected through cross-validation. **(B)** Cross-validation mean squared error (MSE) across different *λ* values. Blue bars represent the mean MSE with error bars indicating standard deviation. The vertical dashed line marks the optimal *λ* value where the model achieved minimal prediction error. **(C)** Final model coefficients for the 14 selected features at optimal *λ*. Horizontal bars show the magnitude and direction of each feature's contribution to feeding intolerance prediction, with positive coefficients (rightward bars) indicating increased risk and negative coefficients (leftward bars) indicating decreased risk.

Cross-validated LASSO regression was employed to identify the optimal set of predictive features by determining the best regularization parameter (*λ*). Figure 1A illustrates the LASSO coefficient paths, showing how feature coefficients progressively shrunk to zero as *λ* increased, enabling effective feature selection. As *λ* increased, feature coefficients progressively shrank to zero, enabling effective feature selection. Cross-validation identified an optimal *λ* value of 0.0095. Figure 1B shows the cross-validation mean squared error (MSE) across different *λ* values, with red dots representing the mean MSE and blue vertical lines indicating the standard deviation. The vertical dashed line marks the optimal *λ* value where the model achieved minimal and stable prediction error, demonstrating robust performance in distinguishing between feeding tolerance and intolerance.

Using the optimal *λ* value of 0.0095, 14 key features were retained in the final LASSO model: invasive mechanical ventilation, time to first feeding, gender, cesarean section, dexamethasone use, gestational diabetes mellitus, premature rupture of membranes, abnormal maternal thyroid function, fetal distress, intrauterine infection, multiple gestation, abnormal amniotic fluid, birth weight, and gestational age. In the final LASSO model (Figure 1C), gestational age and birth weight carry negative coefficients, indicating lower predicted FI probability with increasing values, which is consistent with clinical knowledge. Coefficients for other predictors are reported as predictive weights rather than causal effects within the regularized multivariable context.

### Model performance comparison

Using the 14 features selected by LASSO regression, we developed 11 machine learning classification models: logistic regression (LR), support vector machine (SVM), random forest (RF), extra trees, XGBoost, LightGBM, naive Bayes (NB), AdaBoost, gradient boosting (GB), multilayer perceptron (MLP), and K-nearest neighbors (KNN). Model performance was evaluated using 10-fold cross-validation with a 70% training and 30% testing split (Table 3).

**Table 3:** Comparison of diagnostic performance metrics for different machine learning models.

Model name	Accuracy	AUC (95% CI)	Sensitivity	Specificity
LR				
Training set	0.954	0.981 (0.966–0.996)	0.970	0.937
Testing set	0.901	0.937 (0.890–0.984)	0.870	0.931
NaiveBayes				
Training set	0.932	0.976 (0.960–0.992)	0.939	0.925
Testing set	0.894	0.944 (0.905–0.984)	0.913	0.875
SVM				
Training set	0.975	0.991 (0.980–1.000)	0.982	0.969
Testing set	0.851	0.907 (0.856–0.958)	0.826	0.875
KNN				
Training set	0.932	0.986 (0.979–0.994)	0.927	0.937
Testing set	0.801	0.829 (0.761–0.896)	0.812	0.792
RandomForest				
Training set	0.985	0.999 (0.998–1.000)	0.988	0.981
Testing set	0.922	0.960 (0.928–0.992)	0.870	0.872
ExtraTrees				
Training set	0.985	1.000 (0.999–1.000)	0.976	0.994
Testing set	0.851	0.911 (0.863–0.959)	0.841	0.861
XGBoost				
Training set	0.975	0.997 (0.995–1.000)	0.970	0.981
Testing set	0.943	0.947 (0.908–0.985)	0.928	0.958
LightGBM				
Training set	0.948	0.987 (0.978–0.995)	0.964	0.931
Testing set	0.943	0.954 (0.915–0.993)	0.928	0.958
GradientBoosting				
Training set	0.963	0.989 (0.977–1.000)	0.970	0.956
Testing set	0.943	0.961 (0.929–0.992)	0.928	0.958
AdaBoost				
Training set	0.954	0.990 (0.9832–0.998)	0.970	0.937
Testing set	0.957	0.964 (0.929–1.000)	0.957	0.958
MLP				
Training set	0.954	0.984 (0.969–0.998)	0.964	0.944
Testing set	0.865	0.928 (0.884–0.972)	0.826	0.903

LR, logistic regression; SVM, support vector machine; KNN, K-nearest neighbors; XGBoost, extreme gradient boosting; LightGBM, light gradient boosting machine; AdaBoost, adaptive boosting; MLP, multi-layer perceptron.

Given the clinical importance of accurate FI prediction while avoiding unnecessary interventions, models with balanced sensitivity and specificity were prioritized, with particular attention to minimizing false positives. High AUC and overall accuracy were also essential to ensure robust predictive performance. Therefore, sensitivity and AUC were considered the primary metrics for assessing clinical utility.

On the test set, the AdaBoost model demonstrated superior performance, achieving an accuracy of 0.957 and an AUC of 0.964 (95% CI: 0.929–1.000) (Table 3). The model achieved a sensitivity of 0.957 and a specificity of 0.958, effectively balancing the detection of FI cases while minimizing false positives. Following comprehensive evaluation of all performance metrics and clinical considerations, the AdaBoost model was selected as the optimal predictive model for clinical implementation. Detailed hyperparameters and configuration settings for the AdaBoost model are provided in the Supplementary Materials.

### Clinical application analysis of the optimal model

**Figure 2:**
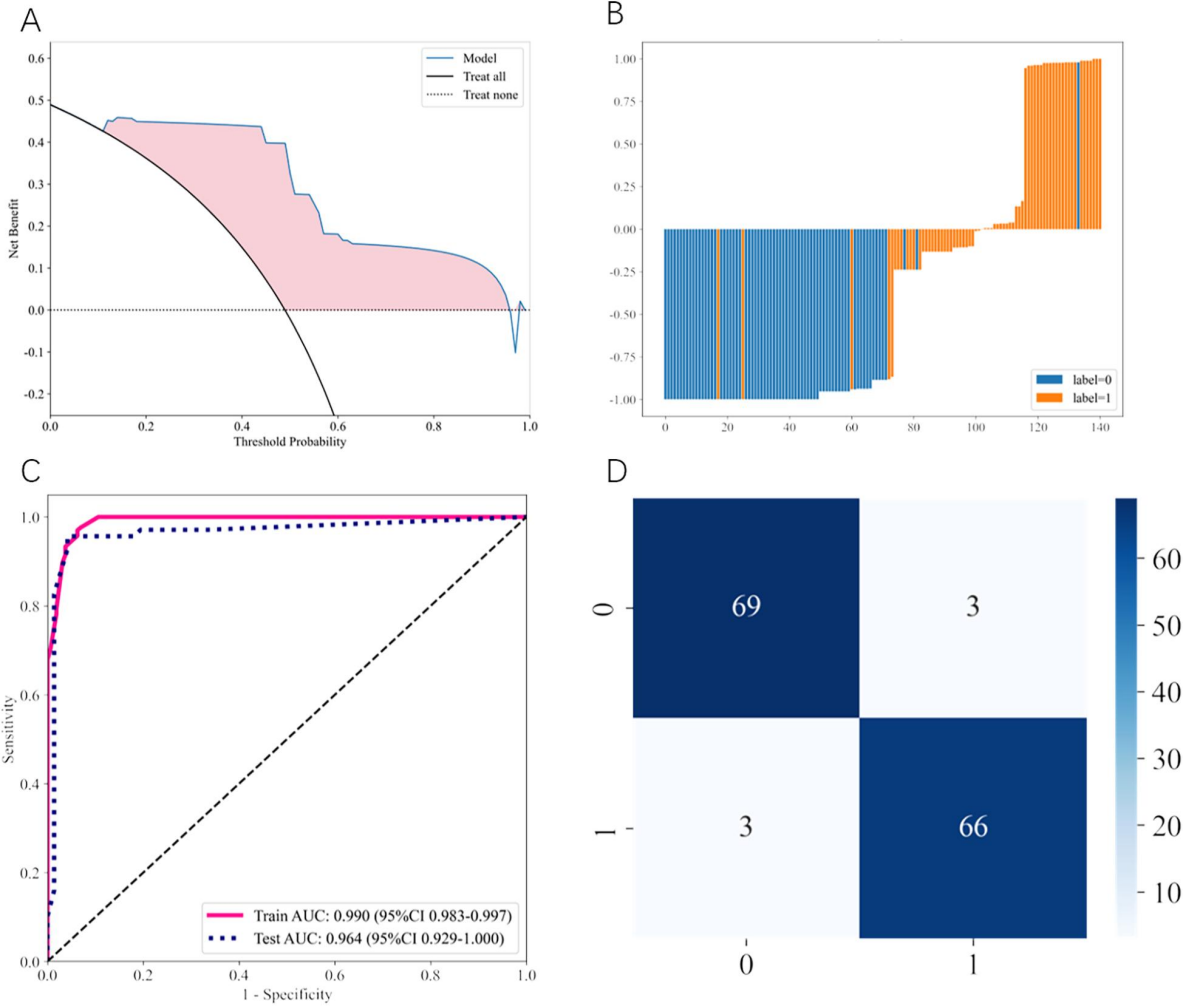
Performance evaluation of the optimal AdaBoost model. **(A)** Decision curve analysis (DCA) comparing the clinical utility of the AdaBoost model (blue line) against “treat all” (black solid line) and “treat none” (black dotted line) strategies. The pink shaded area represents the net benefit gained by using the model across different threshold probabilities. **(B)** Predicted probability distribution histogram for individual patients in the test set. Blue bars represent feeding-tolerant cases (label-0) and orange bars represent feeding intolerance cases (label-1), demonstrating clear separation between the two groups. **(C)** Receiver operating characteristic (ROC) curves showing model performance on training (pink solid line) and test (blue dotted line) datasets. Training AUC: 0.990 (95% CI: 0.983–0.997); Test AUC: 0.964 (95% CI: 0.929–1.000). **(D)** Confusion matrix displaying classification results on the test set. Numbers represent actual counts: 69 true negatives, 3 false positives, 3 false negatives, and 66 true positives.

The comprehensive performance evaluation of the AdaBoost model is illustrated in Figure 2. Decision curve analysis (Figure 2A) demonstrated that the AdaBoost model provided superior net clinical benefit compared to “treat all” or “treat none” strategies across a wide range of threshold probabilities. The predicted probability distribution (Figure 2B) illustrated the risk scores for individual patients, clearly showing the model's discriminative ability between FI and tolerance cases (label-0 and label-1).

**Figure 3:**
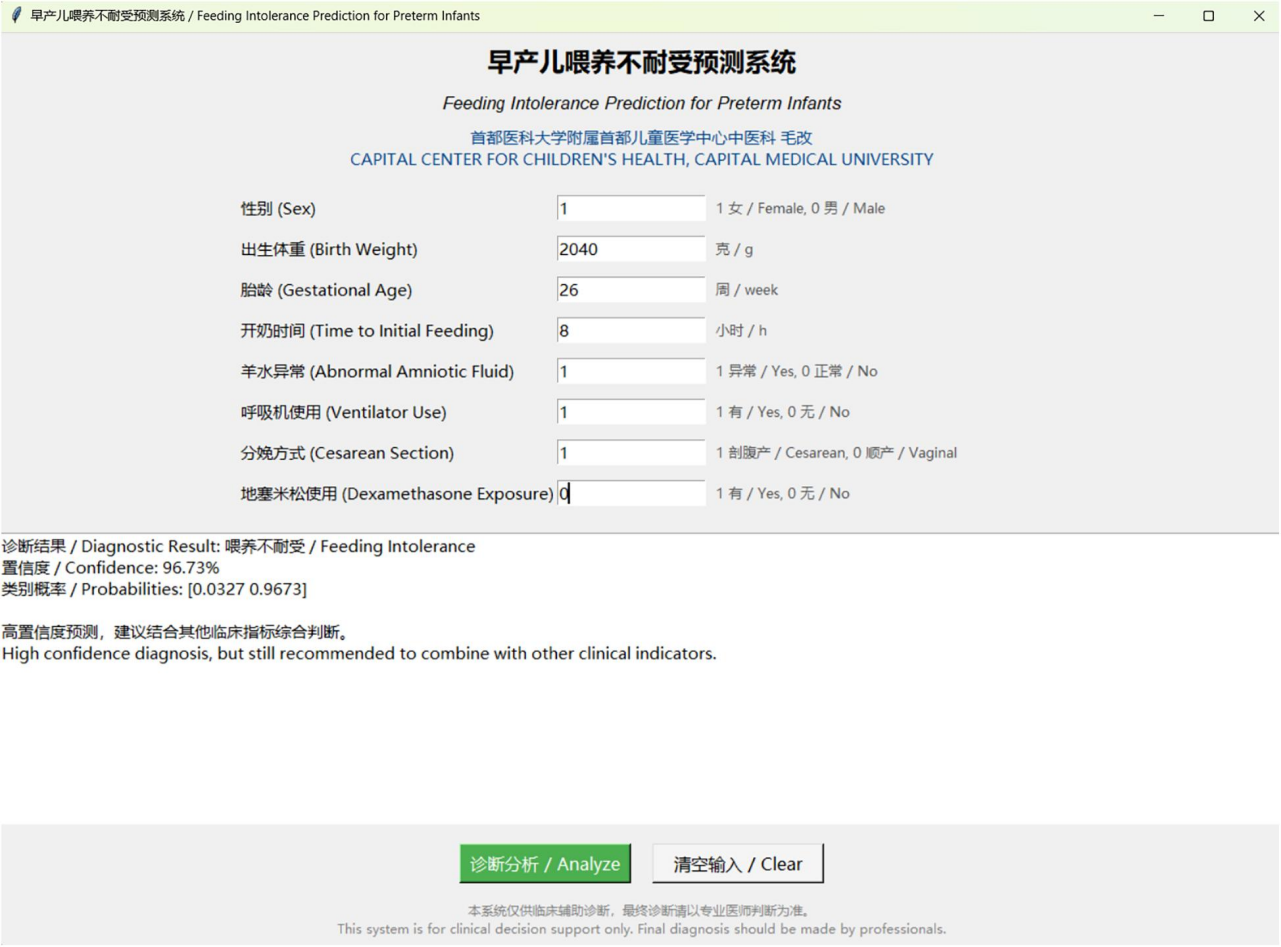
Interactive clinical decision support tool for feeding intolerance prediction. Screenshot of the web-based prediction system showing the user interface for inputting clinical parameters. The tool displays patient information fields including sex, birth weight, gestational age, time to initial feeding, abnormal amniotic fluid, ventilator use, cesarean section, and dexamethasone exposure. The diagnostic result shows a high-confidence prediction of feeding intolerance with 96.73% confidence and probability range [0.0327, 0.9673]. The system provides bilingual support (Chinese and English) and includes clinical decision support recommendations.

The ROC curves (Figure 2C) demonstrate excellent discriminative performance with a training AUC of 0.990 (95% CI: 0.983–0.997) and a test AUC of 0.964 (95% CI: 0.929–1.000), indicating robust model performance without significant overfitting. The confusion matrix (Figure 2D) revealed that the AdaBoost model correctly identified 66 of 69 FI cases and 69 of 72 feeding-tolerant cases in the test set, with only 3 false positives and 3 false negatives, demonstrating strong classification performance.

To facilitate clinical implementation of the AdaBoost model, we developed an interactive prediction tool based on the algorithm. The software features an intuitive interface that allows clinicians to input standard clinical parameters and obtain real-time predictions for FI risk. This tool has the potential to enhance both efficiency and accuracy of FI risk assessment in preterm infants (Figure 3).

## Discussion

This study demonstrates that a machine learning model incorporating 14 routinely available clinical variables can accurately predict feeding intolerance in preterm infants prior to feeding initiation. The AdaBoost model significantly outperformed traditional risk assessment approaches and single-variable predictors, supporting the potential for early, individualized risk stratification for FI.

Gestational age and birth weight emerged as the strongest predictors, which aligns with established international evidence (10). Lower gestational age is associated with immature gastrointestinal motility, reduced digestive enzyme activity, and compromised mucosal barrier function, all of which predispose infants to FI (11). Low birth weight, particularly very low birth weight (<1,500 g), is widely recognized as a risk factor for FI due to its association with prematurity and underdeveloped organ systems (12).

Invasive mechanical ventilation was a strong independent predictor of FI in our model, consistent with findings from relevant research that have documented associations between prolonged respiratory support and delayed enteral feeding, impaired splanchnic perfusion, and altered gut motility (13). Early identification of ventilated infants at high risk for FI may inform targeted feeding protocols and monitoring strategies.

Delayed initiation of enteral feeding was also associated with increased FI risk. International guidelines, including those from ESPGHAN and WHO, recommend early, progressive enteral nutrition for stable preterm infants, and recent large cohort studies demonstrate that feeding initiation within 24–72 h reduces FI and NEC risk without increasing complications (14, 15). Our findings support these recommendations and underscore the importance of timely feeding initiation.

Cesarean section, although associated with altered gut microbiota and increased perinatal morbidity, demonstrated only a modest association with FI in our cohort. International meta-analyses have reported inconsistent findings, suggesting that delivery mode may influence FI primarily through its effects on early microbial colonization rather than as a direct causal mechanism (16, 17).

Dexamethasone exposure, both prenatal and postnatal, was an independent predictor of FI. This finding is consistent with studies linking corticosteroid use to increased risk of gastrointestinal perforation and compromised gut integrity (18). While corticosteroids remain essential for specific clinical indications, enhanced monitoring for FI in exposed infants may be warranted.

Gestational diabetes mellitus was associated with increased FI risk, consistent with global evidence indicating higher rates of neonatal morbidity, altered gut motility, and feeding difficulties in infants of diabetic mothers (19). Premature rupture of membranes and intrauterine infection, including chorioamnionitis, were also significant predictors, reflecting the impact of intrauterine inflammation on gut development and feeding tolerance (20).

Abnormal maternal thyroid function, while less extensively studied, has been suggested to influence fetal gastrointestinal and neurodevelopment, and our findings indicate a potential association warranting further investigation (21).

Multiple gestation was independently associated with FI, though the association was modest. While twins and triplets have higher overall rates of prematurity and morbidity, direct associations with FI are less well-established in the literature (22). Although abnormal amniotic fluid is commonly considered a risk factor for adverse neonatal outcomes, in our model, it was associated with a slightly decreased risk of feeding intolerance. This may be due to confounding by other stronger risk factors (such as prematurity and low birth weight), the impact of multicollinearity among predictors, as well as potential differences in clinical management and intervention for pregnancies complicated by abnormal amniotic fluid. Furthermore, the relatively small number of cases or heterogeneity in the types of amniotic fluid abnormalities may also contribute to this finding. Therefore, this result should be interpreted with caution, and further studies with larger and more diverse cohorts are needed to clarify this association (23). In a multivariable regularization framework, the directions of certain coefficients (e.g., abnormal amniotic fluid and multiple gestation) are not fully aligned with clinical intuition. This phenomenon may relate to conditional (context-dependent) effects, variable heterogeneity, indication bias from clinical management, and the way regularization allocates weights among correlated features. Accordingly, we report these coefficients as predictive weights rather than causal effects; the key directions consistent with clinical consensus (e.g., the protective roles of gestational age and birth weight) are stably reflected in the model.

AdaBoost is an ensemble learning method that sequentially combines multiple weak learners (typically decision trees) to create a strong classifier. The algorithm iteratively adjusts the weights of misclassified samples, forcing subsequent learners to focus on previously difficult cases, thereby improving overall predictive accuracy. This adaptive weighting mechanism makes AdaBoost particularly effective for medical prediction tasks where class imbalance and complex feature interactions are common challenges.

In the context of feeding intolerance prediction, AdaBoost's ability to handle non-linear relationships among clinical variables while maintaining interpretability represents a significant advantage over traditional logistic regression models (24). The algorithm's robustness to overfitting, combined with its capacity to identify subtle patterns in clinical data, likely contributed to its superior performance (AUC: 0.964) compared to other machine learning approaches tested in our study. Furthermore, AdaBoost's inherent feature importance ranking capabilities align well with clinical decision-making processes, allowing healthcare providers to understand which factors most strongly influence FI risk predictions.

The development of an interactive clinical decision support tool represents a crucial step toward translating research findings into clinical practice. Our software application transforms the complex AdaBoost algorithm into an intuitive, user-friendly interface that enables real-time risk assessment at the bedside. This tool addresses a critical gap in neonatal care by providing objective, evidence-based risk stratification before feeding initiation, potentially reducing clinical uncertainty and supporting more informed decision-making.

The clinical significance of this tool extends beyond individual patient care to broader healthcare system implications. By enabling early identification of high-risk infants, the software may facilitate proactive management strategies, including enhanced monitoring protocols, modified feeding approaches, and early consultation with pediatric gastroenterology specialists. This predictive capability could potentially reduce the incidence of severe feeding complications, shorten hospital stays, and improve long-term neurodevelopmental outcomes through optimized nutritional support.

Moreover, the standardization of risk assessment through our decision support tool may reduce inter-clinician variability in FI risk evaluation, particularly valuable in settings with varying levels of neonatal expertise. The tool's integration potential with electronic health records systems could further streamline clinical workflows and ensure consistent application of evidence-based risk prediction across different healthcare settings.

When compared with international literature, our findings demonstrate broad consistency in the direction and magnitude of FI risk factors. However, our study represents one of the first applications of such a comprehensive, pre-feeding machine learning model in a Chinese neonatal population. The model's robust performance, even within a single-center cohort, suggests potential for broader applicability and multicenter validation. The clinical decision support tool derived from our model further facilitates practical, bedside implementation by clinicians.

This study has several limitations, including its retrospective, single-center design and the generalizability of the current model remains to be confirmed due to the lack of external validation on a completely independent dataset, which is crucial for the model's clinical adoption. Some potentially relevant variables, such as detailed gut microbiota profiles or specific prenatal exposures, were not assessed. Future research should focus on prospective, multicenter studies with external validation in diverse populations and incorporation of additional biomarkers when feasible. Ethical considerations regarding the use of artificial intelligence in neonatal care, including data privacy, model interpretability, and healthcare equity, must be addressed through adherence to established international frameworks.

## Conclusion

AdaBoost model incorporating routinely available clinical variables enables accurate prediction of feeding intolerance in preterm infants prior to feeding initiation. This approach demonstrates superior performance compared to conventional risk assessment methods and supports early risk stratification with potential for broader clinical application. Prospective multicenter validation studies are needed to confirm these findings and facilitate clinical implementation.

## Data Availability

The original contributions presented in the study are included in the article/Supplementary Material, further inquiries can be directed to the corresponding author.
