# Supplementary material for: Machine learning prediction of feeding intolerance in preterm infants: a pre-feeding risk stratification model
Source: Front Pediatr. 2025 Sep 5;13:1646973. doi: 10.3389/fped.2025.1646973 (PMC12446353; doi:10.3389/fped.2025.1646973)
Supplement: Supplementary file 1 [file DataSheet.docx]

**Supplementary Material - AdaBoost Model Configuration**

AdaBoost Hyperparameters and Model Specifications

The optimal AdaBoost classifier was configured with the following parameters:

Core Hyperparameters:

n_estimators: 10 (number of weak learners in the ensemble)

learning_rate: 1.0 (shrinkage parameter applied to each classifier)

algorithm: SAMME.R (Real AdaBoost algorithm for probability estimates)

base_estimator: None (default DecisionTreeClassifier with max_depth=1)

random_state: 0 (seed for reproducible results)

Base Estimator Configuration:

Since base_estimator was set to None, the model used the default DecisionTreeClassifier (decision stumps) with the following implicit parameters:

max_depth: 1 (creating decision stumps)

min_samples_split: 2 (default)

min_samples_leaf: 1 (default)

Model Training Details:

Total trained estimators: 10

Target classes: [0, 1] (feeding tolerance vs. feeding intolerance)

Feature subset used: 8 features from the 14 LASSO-selected candidates

Feature Importance Analysis:

The final AdaBoost model assigned the following importance weights to the 8 selected features:

Feature 1 (Sex): 0.0

Feature 2 (Birth weight): 0.2

Feature 3 (Gestational age): 0.5

Feature 4 (Time to initial feeding): 0.1

Feature 5 (Abnormal amniotic fluid): 0.0

Feature 6 (Ventilator use): 0.1

Feature 7 (Cesarean section): 0.0

Feature 8 (Dexamethasone exposure): 0.1

Gestational age demonstrated the highest feature importance (0.5), followed by birth weight (0.2), indicating these variables as the primary drivers of the model's predictive performance.

Technical Environment:

Model trained with: scikit-learn 1.0.2

Model persistence: joblib format

Python environment: Python 3.10

Note: Version compatibility warnings were observed when loading the model with scikit-learn 1.2.2, consistent with documented limitations in model persistence across different scikit-learn versions.

Algorithm Selection Rationale:

The SAMME.R algorithm was selected over SAMME as it provides probability estimates and typically converges faster than the discrete SAMME algorithm, making it more suitable for clinical decision support applications requiring prediction confidence scores.
